# Matrix-Assisted Laser Desorption and Electrospray Ionization Tandem Mass Spectrometry of Microbial and Synthetic Biodegradable Polymers

**DOI:** 10.3390/polym15102356

**Published:** 2023-05-18

**Authors:** Paola Rizzarelli, Marco Rapisarda

**Affiliations:** Institute for Polymers, Composites and Biomaterials, Consiglio Nazionale delle Ricerche (CNR), Via Paolo Gaifami 18, 95126 Catania, Italy; marco.rapisarda@ipcb.cnr.it

**Keywords:** biodegradable polymers, tandem mass spectrometry, multi stage mass spectrometry, MALDI, ESI, characterization, degradation, drug delivery systems

## Abstract

The in-depth structural and compositional investigation of biodegradable polymeric materials, neat or partly degraded, is crucial for their successful applications. Obviously, an exhaustive structural analysis of all synthetic macromolecules is essential in polymer chemistry to confirm the accomplishment of a preparation procedure, identify degradation products originating from side reactions, and monitor chemical–physical properties. Advanced mass spectrometry (MS) techniques have been increasingly applied in biodegradable polymer studies with a relevant role in their further development, valuation, and extension of application fields. However, single-stage MS is not always sufficient to identify unambiguously the polymer structure. Thus, tandem mass spectrometry (MS/MS) has more recently been employed for detailed structure characterization and in degradation and drug release monitoring of polymeric samples, among which are biodegradable polymers. This review aims to run through the investigations carried out by the soft ionization technique matrix-assisted laser desorption/ionization mass spectrometry (MALDI-MS) and electrospray ionization mass spectrometry (ESI-MS) MS/MS in biodegradable polymers and present the resulting information.

## 1. Introduction

Bio-based and biodegradable plastic materials are worthy of supporting a successful conversion to a circular economy [1,2]. Plastic items should be recyclable or biodegradable to avoid their accumulation in the open environment. In the past, different polymers that can fulfill both end-of-life options have been developed and put on the market. The most attractive and extensively used biodegradable polymers, commercially available for their appealing properties, are polylactide (PLA), poly(butyleneadipate-co-butylenetherephthalate) (PBAT), and polyhydroxyalkanoates (PHAs) [3,4,5,6,7]. Growing environmental attention and legislative choices make foreseen a rising share of use and an extension of the application fields. Yet, the widespread usage of biodegradable polymers is restricted by cost and mechanical properties, which are still not competitive with the conventional plastic materials they should substitute. Thus, a remarkable economic effort is still devoted to customizing the chemical–physical properties of biodegradable polymers that depends on the structural ones. Nowadays, there is a high request for developing innovative biodegradable polymer-based items for different areas, i.e., packaging solutions, innovative biomedical applications, and agricultural fields.

Whatever the class of polymers, improving the performance needs a reliable characterization of both physicochemical and mechanical properties. Polymer analysis can include several distinct or interconnected features. Consequently, a detailed characterization of synthetic polymers is essential in outlining the structure–properties relationships and their employment. It is of relevant importance in troubleshooting polymer manufacturing processes or in safety aspects as well as in quality control.

Modern mass spectrometry (MS) can disclose relevant structural specifics in polymer analysis. It has been applied ever more successfully also because of the noteworthy progress in the design of the instruments and advanced configurations as well as data processing tools, as evidenced in articles, reviews, books, and book chapters [8,9,10,11,12,13,14,15,16,17,18,19,20,21,22,23,24,25,26,27,28,29,30]. In fact, MS soft ionization methods, mainly matrix-assisted laser desorption ionization-time of flight MS (MALDI-TOF MS) and electrospray ionization MS (ESI-MS) for their sensitivity and speediness, supply the tiniest structural and architectural details also in complex polymeric systems and biodegradable polymeric materials, composites, and blends as well [15,16,19,28,31,32,33,34,35,36,37]. By ESI and/or MALDI-MS, exhaustive understandings of biodegradable polymers (i.e., polymerization mechanisms, copolymer architecture, residual monomers, drug release, and polymer degradation mechanisms) have been obtained. Most of the MS investigations into polymer systems, including the biodegradable ones, have been carried out using single-stage MS, i.e., detecting the mass-to-charge ratio (*m*/*z*) of unbroken ions. However, single-stage mass data are not always sufficient, for example, to verify definitely a polymer structure or samples in which isobaric or isomeric species are produced. In several instances, tandem MS (MS/MS) can be helpful confirming mass spectra interpretation and providing the supplementary information required [38,39,40,41,42,43]. In MS/MS, chosen precursor ions are induced to undertake chemical reactions, in most cases with unimolecular dissociations, changing their charge or mass. The product ions originated are then mass analyzed. MS/MS has been more and more employed to analyze synthetic polymers. In fact, it can provide significant information on end groups or in-chain functionalization, distinguish isobaric and isomeric chains, differentiate linear and cyclic species, and reveal macromolecular connectivity, sequence distribution, and complex architectures [14,38,39,41,44,45,46,47]. For assertive structure assignments, preliminary studies on the fragmentation pathways are the first step to understanding MS/MS spectra. In fact, the comprehension of the preferred fragmentation processes represents a sort of guideline on how to establish the structure of diagnostic product ions detected in tandem with MS spectra and then go back to the macromolecular structure [40]. The collision-induced dissociation (CID) approach is the most adopted fragmentation tool for the structural characterization of gas-phase ions [38,39,42,44,45,46,47,48,49,50].

**Figure 1:**
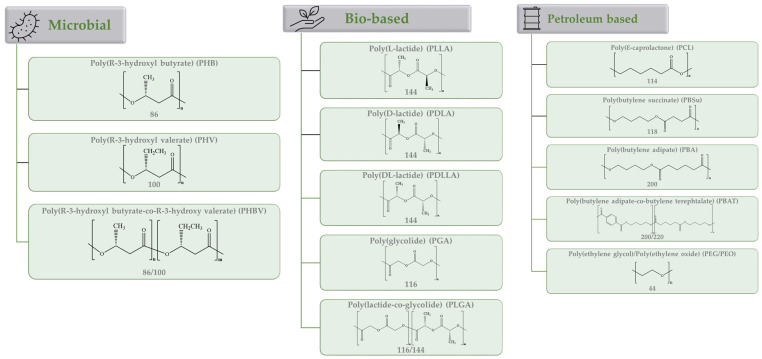
Overview of the most representative microbial and synthetic biodegradable polymers analyzed by MALDI and ESI-MS and MS/MS.

Several types of biodegradable polymers have been examined by MALDI-MS, ESI-MS, and MS/MS. Figure 1 shows an overview of the most representative microbial and synthetic biodegradable polymers studied by MALDI and ESI-MS, and MS/MS. In the last decades, studies on fragmentation mechanisms [47,48,49,50,51,52,53,54,55], structure and architecture [56,57,58,59,60,61,62,63,64,65,66,67,68,69,70,71,72,73,74,75,76,77,78,79,80,81,82,83,84,85,86,87,88], biopharmaceutical and drug delivery systems (DDS) [89,90,91,92,93,94,95,96,97,98,99,100,101,102,103,104,105,106,107,108,109,110], and polymer degradation products [111,112,113,114,115,116,117,118,119,120] have been performed. Frequently with the support of other analytical techniques (mainly nuclear magnetic resonance, NMR, and liquid chromatography, LC), relevant information on architectures and structural details of the biodegradable polymer [61,63,65,66,67,68,69,70,71,72,74,76,77,80,81,82,84,121,122,123,124,125,126], end-groups [58,61,62,64,67,70,72,74,77], copolymer composition [55,57,59,60,62,74,78,83,84] and sequence distribution [47,49,49,54,58,61,74,76,78,80], pharmacokinetic, the fate of polymeric excipients, DDS [98,103,104,105,106,107,108,109,110] and degradation products and mechanisms [111,112,113,114,115,116,117,118,119,120] have been gained.

This review aims to summarize the studies carried out by MALDI and ESI-MS/MS on biodegradable polymers, underlining the information supplied and the analytical methodology designated. Inspection of the literature shows that in most cases, MALDI and ESI-MS/MS have been utilized for the structural characterization of biodegradable polymers, less on the evaluation of their degradation behavior. The ESI source, CID tool, and positive ion mode are prevalently chosen, even if the fragmentation pathways of the anions could be easier than those of the corresponding cations [127]. More recently, several studies have been focused on DDS and pharmacokinetics by MS/MS, thanks to the technological progress and combining ESI with separation techniques. Overall, MS has been more extensively employed in the analyses of biodegradable polymers [14,15,16,19,21,23,25,28,31,32,33,34,35,36,37,44,128] than tandem MS [38,39,40,41,42,43,45], even if MS/MS is highly informative. This should be reasonably due to the time-consuming step related to the data processing and interpretation in MS/MS [14,15,19,28,39,42], even though tandem MS analysis has provided valuable information on biodegradable polymer architectures, end-group functionalities, bond connectivity, copolymer sequences, etc. (Table 1).

## 2. MALDI Tandem Mass Spectrometry Studies

Due to the developed technologies and analytical strategies, MALDI-TOF MS has become a normal analytical method for the analysis of synthetic polymers, supplying complete structural information with high sensitivity [17,21]. It has been fruitfully utilized in both characterization and degradation investigations. Well-known weaknesses in the determination of the molecular weight, especially when dealing with high mass range, are related to the polydisperse nature of polymers [129]. Furthermore, through the so-called post-source decay (PSD), it is feasible to acquire fragment ion spectra even though MALDI is a soft ionization technique [130]. The progress in the design of the MALDI-TOF/TOF instruments, to improve the experimental procedures, acquiring more consistent information, avoided many restrictions of the PSD and CID in a common MALDI instrument. In a MALDI-TOF/TOF mass spectrometer operating in tandem MS mode (MALDI-TOF/TOF-MS/MS), the high-speed ions separated in the first TOF analyzer are selectively transmitted by a timed ion gate into the collision cell and undergo fragmentation via CID. Fragment ions (product ions) from the precursor are transmitted and accelerated into the second TOF mass analyzer, where further mass separation occurs. Finally, separated product ions reach the detector, and MS/MS spectra are recorded [131]. MALDI-MS/MS has been used for the unambiguous analysis of polymer components providing detailed and helpful information to establish bond sequences, check synthetic procedures and undesired side reactions, or differentiate isobaric species. In order to solve some more complex cases, strategies of data processing and analytical methods had to be also developed by coupling LC or ion mobility (IM) with soft ionization tandem MS/MS [62,66,69,76,77,78,79].

MALDI-TOF/TOF-MS/MS has been well used to study biodegradable polymer samples, such as polyesters and polyesteramides [19,38]. It disclosed structural information concerning the sequence of ester and amide bonds in synthetic polyesteramides [47,49,54] and the fragmentation mechanisms in biodegradable polyesters [48,52]. Fragmentation studies have been carried out on biodegradable aliphatic polyesters [48,52] and polyesteramides [47,49,54] by MALDI CID experiments. Selected Na^+^ adducts of poly(butylene adipate) (PBA) oligomers bearing different chain ends were analyzed by MALDI/TOF-TOF CID using air as the collision gas. The same series of product ions were detected whatever the end groups of the precursor ions. Then, three major fragmentation pathways were suggested for PBA involving the cleavage of –O–CH_2_-bonds through a β-H transfer rearrangement, -CH_2_–CH_2_- (β–β) bonds in the adipate moiety, and ester bonds [48]. The β-H transfer rearrangement is a classic thermal degradation mechanism in polyesters, and it involves the transfer of hydrogen in the β position to the carboxyl group of the diacid via a six-membered cyclic transition state [29]. Additional fragmentation pathways were revealed in the MALDI-TOF/TOF-MS/MS study of poly(butylene succinate) (PBSu). The Na^+^ adduct ions of cyclic and linear oligomers with diverse end groups were selected as precursor ions. MS/MS spectra were acquired with and without the collision gas (argon). In addition to the pathways detected in the investigation on PBA, two fragmentation mechanisms were suggested and related to the end-groups of the precursor ions. In particular, the revealing of succinic anhydride was associated with the precursor ions bearing succinic acid end-groups. While the detection of macrocyclic oligoesters was due to terminal hydroxyl groups, and in all probability, they were originally from an intramolecular transesterification mechanism. The β-H transfer rearrangement was established as the most favored fragmentation pathway since the only one taking place in PBSu in the absence of the collision gas. In contrast, in the MALDI CID experiments using argon as the collision gas, almost all types of bonds were broken. Six fragmentation pathways were identified when using argon as the collision gas in relation to the assigned structures of the most abundant product ions [52].

**Figure 2:**
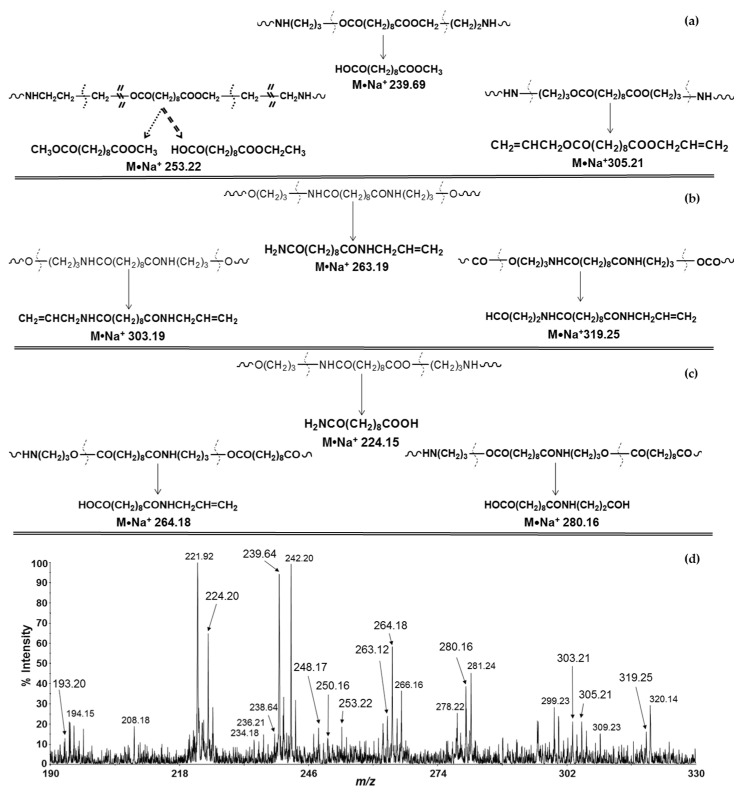
Bond cleavages leading to the diagnostic product ions from (**a**) ester/ester, (**b**) amide/amide, and (**c**) ester/amide sequences. (**d**) Enlarged portion of MALDI-TOF/TOF-MS/MS spectrum of the sodiated cyclic oligomers at *m*/*z* 1228.8 of the PEA-Pro sample (collision gas = argon) (reprinted from [54]).

PSD and MALDI-CID analysis with a TOF-TOF instrument was useful in the fragmentation studies of polyesteramide (PEAm). Moreover, MALDI-TOF/TOF-MS/MS was successfully applied in the determination of the sequence of the ester, and the amide bonds as well as the structure elucidation of side products originated during the polymerization reaction in PEAm samples [47,49,54]. In particular, PSD and MALDI CID have been used to characterize PEAm synthesized through a melt condensation of sebacic acid and 4-amino-1-butanol. The Na^+^ adduct ions of PEAm cyclic and linear species bearing dicarboxyl groups, carboxyl and hydroxyl groups, and diamino alcohol groups were selected as precursor ions. MS/MS data were acquired in the absence and presence of a collision gas (air or argon). Different end groups had no effects on the fragmentation of Na^+^ PEAm oligomers, similar to PBA [48]. Two main fragmentation patterns occurred, involving the scission of the -O–CH_2_- bonds via a β-H transfer rearrangement and the -CH_2_–CH_2_- (β–γ) bond cleavages in the sebacate moiety. In the MALDI CID spectra recorded using argon as the collision gas of linear oligomeric chains terminated by diamino alcohol groups and cyclic oligomers, product ions detected in the low-mass range were diagnostic to prove a random distribution of ester and amide bond sequences in the polyesteramide sample. Moreover, tandem MS analysis yielded valuable information to elucidate the structures of precursor ions resulting from side reactions during the synthetic procedure [49]. Very recently, Rizzarelli et al. studied biodegradable PEAm samples based on sebacic acid and 3-amino-1-propanol by NMR, MALDI-TOF/TOF-MS/MS, thermogravimetric analysis (TGA), and pyrolysis-gas chromatography/mass spectrometry (Py-GC/MS). Again, insights on the fragmentation mechanisms and ester/amide bond sequences were achieved by MALDI-TOF/TOF tandem MS analysis accomplished on Na^+^ adducts of cyclic and linear oligomers. All the MALDI-TOF/TOF-MS/MS spectra acquired showed a similar (most abundant) series of product ions. Then, different chain end groups did not affect the fragmentation pathways. As in similar works [47,48,49,52], the β-hydrogen transfer rearrangement was confirmed as the main fragmentation mechanism. An expanded portion (mass range *m*/*z* 190–330) of the MALDI-CID spectrum of the Na^+^ cyclic oligomers at *m*/*z* 1228.8 is reported in Figure 2. Based on the structures of some diagnostic ions, the authors showed that the orientation of 3-amino-1-propanol along the macromolecular chain is random. In fact, the ions detected at *m*/*z* 193, 239, 305, and 253 were indicative of ester/ester sequences (Figure 2a), while those at *m*/*z* 263, 319, and 303 derived from amide/amide sequences (Figure 2b). Finally, the structures of the product ions at *m*/*z* 224, 250, 264, 248, and 280 were related to ester/amide sequences (Figure 2c).

**Figure 3:**
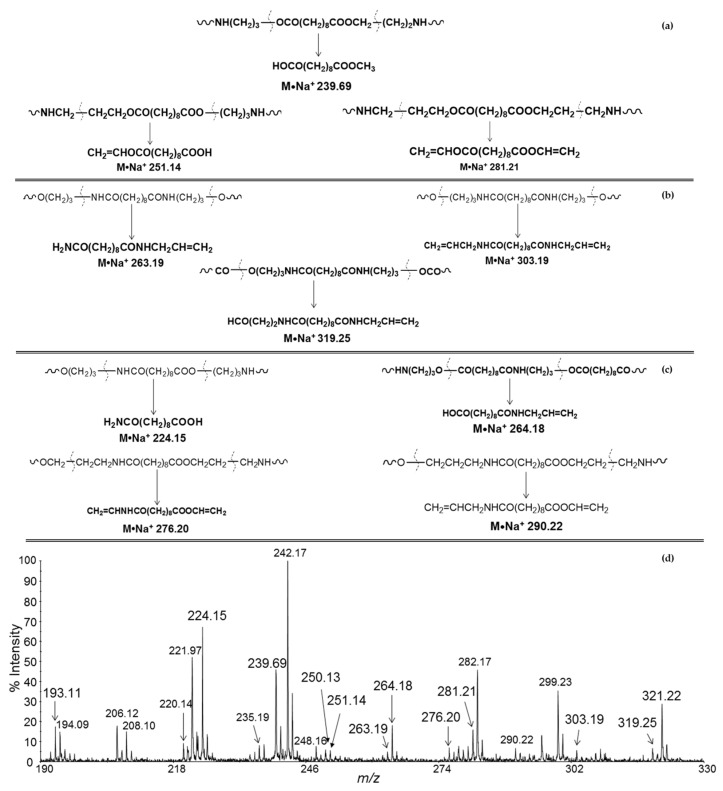
Bond cleavages leading to the diagnostic product ions from (**a**) ester/ester, (**b**) amide/amide, and (**c**) ester/amide sequences. (**d**) Enlarged portion of MALDI-TOF/TOF-MS/MS spectrum of the sodiated dicarboxyl-terminated oligomers at *m*/*z* 1303.9 of the PEA-Pro sample (collision gas = argon) (reprinted from [54]).

Similarly, MALDI-CID experiments performed on the Na^+^ diamino-alcohol-ended chains at *m*/*z* 1303.9 confirmed that the ester and amide bonds were distributed casually in the polymer chains. Figure 3 shows an expanded portion of the MS/MS spectrum (mass range *m*/*z* 190–330). In such cases, additional Na^+^ product ions derived from (a) ester/ester, (b) amide/amide, and (c) ester/amide sequences were identified [54].

MALDI-TOF/TOF-MS/MS was also used by Ashby et al. to check the structure of microbial poly-3-hydroxybutyrate-*co*-3-hydroxyvalerate (PHBV) block copolymers produced by *Burkholderia sacchari* using xylose and levulinic acid as carbon sources. By ^13^C NMR, the authors established the presence of high concentrations of 3HB-3HB and 3HV-3HV homopolymeric dyads; by MS, performed on partially hydrolyzed products, block sequences, not fitting to the Bernoullian statistics for randomness, were revealed. Nevertheless, MALDI-TOF/TOF tandem analysis of selected oligomers displayed the mass-loss of 86 amu (a 3HB unit) and 100 amu (a 3HV unit), indicating some randomness within the polymer chains [83].

Structural analyses of cyclodextrin–oligoester derivatives have been carried out by MALDI-MS, with the support of NMR and tandem MS [80,87,88]. Blaj et al. studied the influence of different reaction conditions on the β-CD ring-opening oligomerization (ROO) of D,L-lactide by MALDI. They carried out an in-depth analysis of cyclodextrin–oligolactide conjugates (CDLA) and degradation products formed during the synthesis procedure using an optimized MALDI-MS characterization method. They validated MALDI-MS kinetics by NMR spectroscopy. The MS characterization accurately depicted the evolution of the CDLA products throughout the ROO reaction. They obtained information about the balance between polymerization and depolymerization processes, which is valuable to avoid undesirable reactions through changing experimental conditions. The MALDI mass spectra highlighted changes in molecular weight (MW) and the presence of unexpected products when diverse solvents and temperatures were employed for the synthesis of CDLA. They demonstrated via MS and fragmentation MS/MS experiments the secondary degradation processes of the employed solvents (DMF or NMP). The cleavage of the DMF and NMP amide bonds generated amines, which played the role of nucleophile activators in the OH-initiated ring-opening polymerization (ROP) of LA [87]. In a similar way, Peptu et al. investigated the solution ROP of ε-caprolactone (CL) in the presence of β-CD, leading to highly water-soluble oligoCL cyclodextrin derivatives (CDCL). Secondary reactions involving the solvent used were revealed by the chemical modification of the CDCL verified using MALDI-MS and MS/MS analyses [88].

The successful functionalization of biodegradable polymer conjugates has been checked by MALDI, ESI-MS, MS/MS, and ion mobility MS (IM-MS) [70,71,75,76,78]. Various studies have also been performed on bioconjugates, hybrid materials constituted by biomolecules connected by covalent bonds to synthetic polymers, with poly(ethylene glycol) (PEG) being the most frequently utilized. The covalent link of PEG to other molecules is named PEGylation, and it is principally employed to prepare peptide and protein drugs. PEGylated drugs are used for therapeutic purposes for the cure of numerous chronic diseases. This class of bioconjugates generally cannot be prepared with elevated purity. NMR and other traditional spectroscopic techniques cannot provide as sufficient structural characterization as the MS methods, combined with separation systems and MS/MS fragmentation, allowed to do [40]. IM-MS offers additional separation efficacy and shape/size selectivity merging dispersion in relation to *m*/*z* (MS dimension) with collision cross section (CCS) and charge (IM dimension). The combination of a soft ionization method (i.e., MALDI or ESI) with IM-MS and MS/MS fragmentation, creating a multidimensional technique, has succeeded in providing noteworthy insights concerning the composition, structure, and architecture of bioconjugates and other complex biomacromolecules. Alawiat et al., by tandem MS and IM-MS/MS techniques, investigated the sequence, derivatization site, and conformation of two alanine-rich peptides and their conjugates with PEG. MALDI-MS, ESI-MS, and tandem MS disclosed the sequence and conformation of polypeptides and bioconjugates with PEG fragmentation. Both MALDI and ESI-MS/MS fragmentation studies revealed that PEG was linked to the C-terminus of the peptides [76]. Furthermore, the same multidimensional MS method has been successfully utilized to clarify the composition, end group structures, chain sequence, and isomeric purity of two copolyesters poly(propylene maleate) (PPM) and poly(propylene fumarate) (PPF). Noteworthy, Sallam et al. proved that the catalyst used in the polymerization process was linked to the macromolecular chains as the initiating polymer end and that the PPM to PPF isomerization, caused by an amine base, proceeded with a quantitative yield [77].

**Scheme 1:**
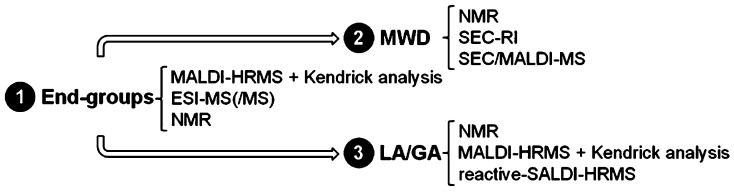
Workflow Summarizing the MS-Based Analytical Strategy Developed for Full Characterization of PLGA Copolymers (reprinted from [84] with kind permission of ACS).

Recently, Fouquet et al. [84] developed an analytical strategy based on MS and MS/MS for a full characterization concerning end group structures, molecular weight, and co-monomeric composition determination of poly(lactide-*co*-glycolide) (PLGA) samples (Scheme 1).

**Figure 4:**
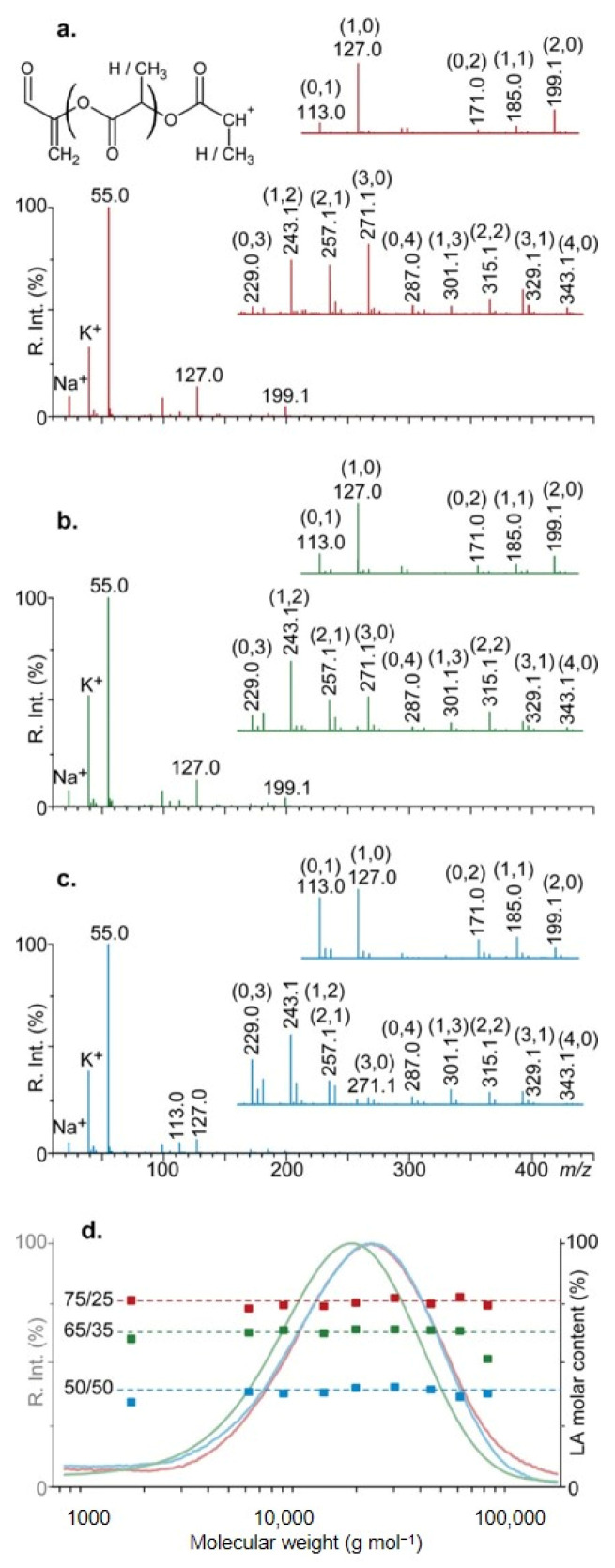
Laser DIUTHAME−spiralTOF mass spectra of (**a**) sample #2 (PLGA 75/25), (**b**) sample #3 (PLGA 65/35), and (**c**) sample #4 (PLGA 50/50), showing in-source fragments of the form C_3_H_3_O(C_3_H_4_O_2_)_x_(C_2_H_2_O_2_)*_y_*^+^, with *x* and *y* the number of intact LA and GA units, respectively. (**d**) LA molar content (%) calculated from product ion abundances measured for unfractionated samples (dotted lines in red for sample #2, green for sample #3, and blue for sample #4) and for nine fractions (colored squares) collected throughout the SEC profile of the three PLGA copolymer samples displayed in the background (reprinted from [84] with kind permission of ACS).

The study was propaedeutic to establish the biodegradability of the four commercial PLGA samples, with different GA molar content (0–50%) and molar mass values (Mw 18 ÷ 75 kg mol^−1^). Size exclusion chromatography (SEC) and NMR were applied as either complementary or validation methods. MALDI was devoted as a favored method to escape the complex distribution of multiply charged ions in ESI spectra. Tandem MS confirmed that cyclic species were most abundant in the low *m*/*z* range. End group structures of linear species deduced from high-resolution (HR) MS analyses agreed with those revealed by NMR. Data on chain ends were crucial to obtain MW data from NMR and/or assess the LA/GA composition from MS. However, the acquisition of additional CID ESI-MS/MS spectra was necessary for the unequivocal identification of the end groups. In order to reduce the step of HRMS data interpretation, particularly challenging in the instance of copolymer samples, Kendrick analysis was systematically applied to MALDI-HRMS. Thus, the constructed 2D plots provided a simplified visualization of the data and allowed the finest proof of less abundant components, ensuring the assignment of all the species. The mass spectra of all four samples showed distributions of Na^+^ macrocycle species, usually detected as by-products in condensation polymerizations [33] and whose MALDI ionization yield is well known to be better than the linear ones [132]. Furthermore, by the end group analysis, copolymer composition was obtained from MS data, using 2D maps and Kendrick analysis of MALDI spectra of both entire and fractionated samples to evaluate composition changes as a function of copolymeric chain length. To investigate LA/GA composition, the Authors employed a surface-assisted laser/desorption ionization (SALDI) soft ionization method called “desorption ionization using through-hole alumina membrane” (DIUTHAME) [133] that induces a widespread CID-like breakup in the lack of a cationizing agent. Fouquet et al. first applied the reactive-SALDI method to PLA homopolymer and, in agreement with the fragmentation pattern obtained, assigned the small product ions (*m*/*z* < 400) of the unfractionated PLGA samples to C_3_H_3_O-(C_3_H_4_O_2_)_x_(C_2_H_2_O_2_)_y_^+^ (Figure 4a–c). Remarkably, the relative abundances of these fragments were dissimilar and related to the initial PLGA composition, more evident in the insets of Figure 4a–c. LA percentage of ∼75, 65, and 40 was determined, being in good agreement with values predictable for the samples analyzed, i.e., #2 PLGA 75/25, #3 PLGA 65/35, and #4 PLGA 50/50, respectively. Reactive-SALDI mass spectra recorded for SEC fractions (Figure 4d) showed similar efficacy of the in-source dissociation process irrespective of chain length [84].

## 3. ESI Tandem Mass Spectrometry Studies

ESI tandem MS has been used more extensively than MALDI-MS/MS in the analysis of biodegradable polymers. In the literature, ESI-MS/MS studies concern fragmentation analysis and insights on comparative dissociation tools [50,51,53,55,82], structural characterization [19,55,63,64,65,67,68,70,71,72,74,75,80,81,83,86,121,122,123,124,125,126], drug delivery systems [89,90,91,95,96,97,98,100,101,102,103,104,105,106,107,108,109,110,111,112] and degradation studies [111,112,113,114,115,116,117,118,119,120] with the significant advantage of possible quantitative analysis, in most case when coupled with liquid chromatography.

### 3.1. Fragmentation Analysis and Dissociation Tools

**Figure 5:**
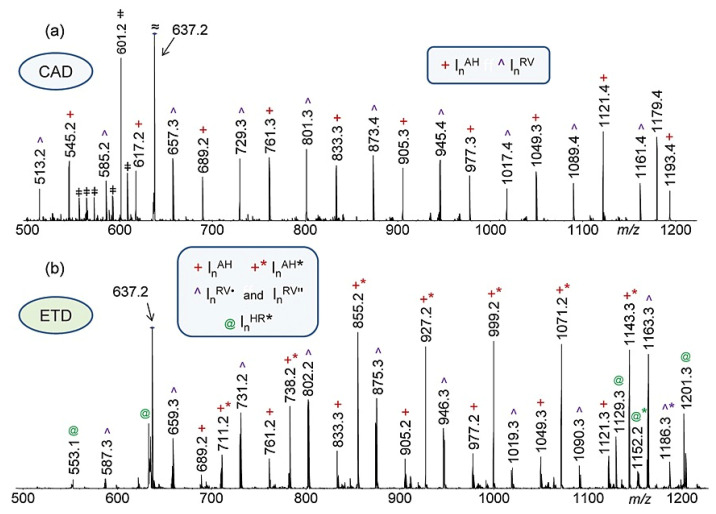
(**a**) CAD and (**b**) ETD MS^2^ spectra of the [M+2Na]^2+^ ion from the poly(lactide) 16-mer (*m*/*z* 637). The notation l_n_ indicates linear fragments with n repeat units; the superscript gives the end groups (see text). (**a**) All CAD fragments are singly charged except for those marked by ‡, which carry 2+ charges (*m*/*z* 556.2, 565.2, 572.2, 592.2 and 601.2). (**b**) All ETD fragments are singly charged; l_n_* denotes doubly sodiated monocations, whereas the notations l_n_^●^ and l_n_^″^ for vinyl-terminated fragments indicate the presence of 1 or 2 additional H atoms, respectively (both are present) (reprinted from [51] with kind permission of Wiley).

Preliminary fragmentation studies and comparative investigations on different dissociation modes have been performed by ESI tandem MS. De Winter et al. [50] carried out by ESI-MS/MS a mechanistic study on the low kinetic energy CID response of diverse Na^+^ adducts of PLA oligomers end-group-modified. Together with the foreseeable fragmentation mechanisms, an investigation of several PLA precursor ions revealed that end group-specific dissociations occurred. Fragmentation pathways followed a favorable six-membered ring transition state (McLafferty-like rearrangement). Sequential and competitive dissociations were also established due to gradual breakages of the oligomers from both ends of the chain. Moreover, the PLA Na^+^ adducts were involved in two consecutive backbone breaks leading to dimer and trimer Na^+^ cations that, in the end, produced a monomeric residue, i.e., the loss of neutral acrylic acid. A theoretical study supported the experiments [50]. Electron-transfer dissociation (ETD) was selected and tested as a complementary ESI-MS/MS method by Scionti and Wesdemiotis. The biodegradable polymers studied included poly(ethylene adipate) and PBA, PLA, and two copolymers. The ETD MS/MS method was compared with those obtained by the classical collision-activated dissociation (CAD) one selecting the same precursor ions. In Figure 5, the (a) CAD and (b) ETD MS^2^ spectra of the [M + 2Na]^2+^ ion from the PLA 16-mer (*m*/*z* 637) are reported [51].

**Scheme 2:**
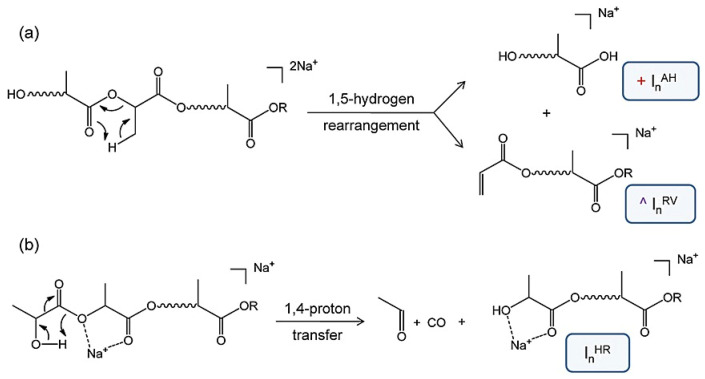
CAD pathways of sodiated poly(lactide) proceeding via (**a**) charge-remote 1,5-H rearrangement at the ester groups and (**b**) charge-induced 1,4-proton transfer at the hydroxy-terminated repeat unit; R = CH_2_CH_2_OCH_3_. The former reaction yields fragments with acid/hydroxy (l_n_^AH^) and ester/vinyl (l_n_^RV^) end groups and the latter truncated chains with the same end groups as the precursor ion (ester/hydroxy, l_n_^HR^) (reprinted from [51] with kind permission of Wiley).

Two distinct series of fragment ions with a difference of 72 Da, corresponding to the PLA repeat unit (C_3_H_4_O_2_), were detected. In the CAD spectrum, all ions were due to linear structure (symbolized by ln, with n repeat units). Different end groups were identified, and letter superscripts were used to label them (A designated a carboxylic acid, H a hydroxy or hydroxyalkyl functionality, R an ester substituent, and V a vinyl or terminal alkene group). Essentially, all fragments derived from a random charge-remote fragmentation along the polymer chain, involving charge-remote 1,5-H rearrangements over the ester groups and the consequent breakages at the (CO)O–alkyl bonds (Scheme 2a).

**Figure 6:**
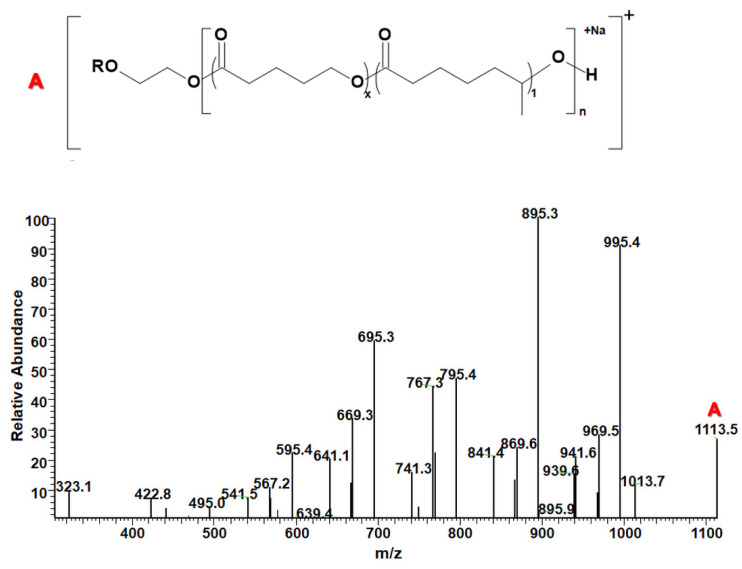
ESI-MS/MS spectrum of the sodium adducts of poly (δ-valerolactone–6-ε-methylcaprolactone) macromolecules and containing one mCL comonomer unit at *m*/*z* 1113. “A” symbolizes the structure of the precursor ion bearing ethylene glycol as an end group (R = H) (adapted from [55]).

**Scheme 3:**
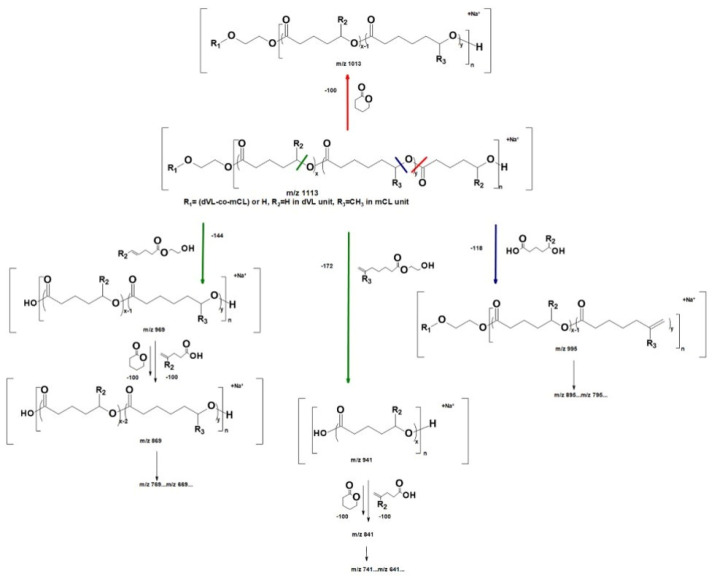
Possible fragmentation pathways for sodium adducts of poly (δ-valerolactone–6-methyl-ε-caprolactone) macromolecules at *m*/*z* 1113; R_1_ = P(dVL-*co*-mCL) or H, R_2_ = H in dVL unit, R_3_ = CH_3_ in mCL unit (reprinted from [55]).

On the contrary, ETD gave rise to a radical anion at the site of electron attachment, whose dissociation occurred by radical-induced cleavage of the (CO)O–alkyl bonds and intramolecular nucleophilic substitution at the (CO)–O bonds (Scheme 2b). One of the main advantages of ETD highlighted was that no sequential fragmentations were stimulated at any substantial level in comparison with the conventional CAD method. This simplified the assignment of mass spectra, allowing an easier and correct interpretation of the end group structures [51]. A similar conclusion was depicted by Prian et al., that compared CID and ETD mass spectra of various lithium adducts ([M + 2Li]^2+^, [M + 3Li]^3+^) of poly(caprolactone) diol (PCL), poly(tetrahydrofurane) (PTHF), and one triblock copolymer (PCL-PTHF-PCL). For both PCL and PTHF homopolymers, the CID of triply lithiated precursor ions produced complex mass spectra compared to the ETD ones, which were simple. In fact, ETD predominantly led to singly charged fragment ions, while CID product ions showed multiple charge states. Furthermore, both MS/MS analyses on the commercial triblock copolymer disclosed that at least a diblock rather than triblock polymer constituted a part of the sample [53]. ESI tandem MS via CID was preliminarily used to establish the fragmentation pattern of telechelic poly(δ-valerolactone–co–6-methyl-ε-caprolactone) oligoesters (P(dVL-co-mCL)), obtained by boric acid biocatalyzed ROP of δ-valerolactone using ethylene glycol and 6-methy-ε-caprolactone as the initiator and the comonomer, respectively. The ESI-MS/MS spectrum of the Na^+^ adducts of OH terminated P(dVL-co-mCL) at *m*/*z* 1113 (named series A) is reported in Figure 6. The MS/MS experiments on the precursor ions at *m*/*z* 1113 highlighted that it was breaking from both chain ends following four sets of fragment pathways. The first series proceeded via the attack of OH end groups on adjacent carbonyl carbon. After that, successive loss of cyclic 5-valerolactone (100 Da) (Scheme 3) occurred through a backbiting reaction arising from the release of uncharged cyclic *δ*-valerolactone of 4-pentenoic acid, previously observed [82]. Then, it appeared that the breaking up stopped as no further loss from the *m*/*z* 1013 product ion was detected. The ions at *m*/*z* 995, due to the second series, originated from the loss of 5-hydroxyvaleric acid (118 Da). Further mass differences of 118 Da were not revealed, but the loss of 100 Da, due to neutral *δ*-valerolactone or 4-pentenoic acid, was detected at *m*/*z* 895, 795, 695, 595, and 495. Random breakage of ester bonds along the polyester chain was observed as well, with the formation of 2-hydroxyethylpentene-4-enoate (144 Da) and the loss of 2-hydroxyethyl 5-methylhex-5-enoate (172 Da). Moreover, additional fragments of neutral ions were found at *m*/*z* 841, 741, 641, 541, and 441 in the same series (Scheme 3) [55].

### 3.2. Structural Characterization

**Figure 7:**
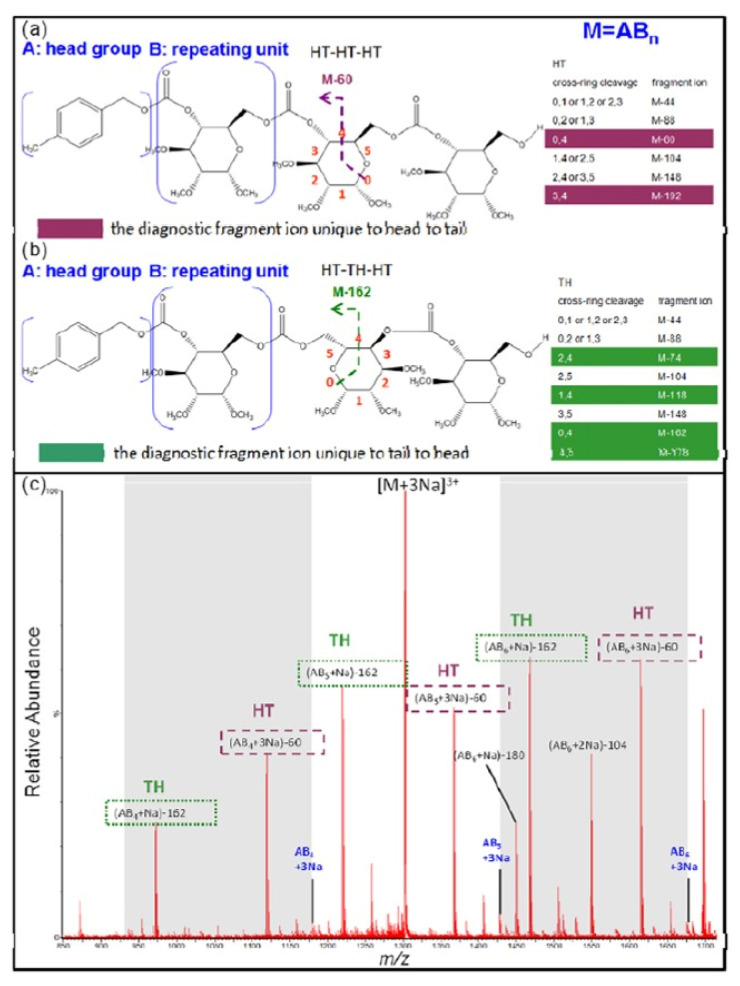
Tandem MS analysis by ETD. (**a**) Schematic of all the possible fragment ions with cross-ring cleavages for a head-to-tail (HT) subunit orientation. M-60 and M-192 are the diagnostic fragment ions for HT orientation. (**b**) Schematic of all the possible fragment ions with cross-ring cleavages for a tail-to-head (TH) subunit orientation. M-74, M-118, M-162 and M-178 are the diagnostic fragment ions for TH orientation. (**c**) ETD MS/MS spectrum of tri-sodiated 15 mer ([AB15 + 3Na]^3+^, *m*/*z* 1305). Note, at each subunit dissociation diagnostic fragment ions of both subunit orientations are present (reprinted from [86] with kind permission of ACS).

ESI-MS/MS has been widely utilized to check the structure of several biodegradable polymeric systems [19,63,64,65,68,71,80,86], in particular for end-groups, degree of purity, bond sequences [19,63,64,65,68], and to investigate the structure of several polymer conjugates [63,65,66,67,71], some of them prepared by ROO [67,80]. Noteworthy, Mikami et al. determined by tandem MS ESI-MS analysis using ETD the regiochemistry of polycarbonates (PC) derived from glucose via an organocatalytic approach. ESI single-stage MS produced two series of ions corresponding to the PC polymer with two and three Na attached. The tri-sodiated 15-mer and 16-mer, [M + 3Na]^3+^, were selected in the mass analyzer and dissociated to obtain predominantly singly charged product ions. Mass spectra assignments were carried out by defining all possible cross-ring breaks for both the head-to-tail (HT) and tail-to-head (TH) orientation of the polymer subunit, formulating a table for each orientation (Figure 7a,b). Each HT and TH orientation originated distinctive cross-ring cleavage ions that were diagnostic to determine regiochemistry for a specific subunit. The ETD fragment ion MS spectrum of tri-sodiated 15 mer (Figure 6) highlighted the removal of whole subunits and product ions with cross-ring scissions between each full subunit loss. The characteristic ions for both HT and TH were observed for each subunit in the ESI-MS/MS spectrum, showing that both orientations exist in each subunit. Thus, the results obtained by ETD tandem MS analysis confirmed that the polymerization generated a random orientation with all three possible regiochemistries (HH, HT, and TT) [86].

Josse et al. verified by an MS/MS procedure the degree of purity accomplished in the cyclization reaction of a linear PLA made by Cu-catalyzed alkyne-azide cycloaddition. Remarkably, the optimized ESI-MS/MS approach showed the presence of trace amounts (<5%) of the linear chains, thanks to radically dissimilar CID behaviors. On the contrary, the traditional techniques (^1^H NMR and single-stage MS) were powerless to detect a residue of the starting material. The developed analytical method could be extended and potentially compliant with other isomeric macromolecules with different fragmentation behavior [68].

Both NMR and ESI-MS/MS were used to confirm the achievement of the procedures employed to synthesize pesticide [63,121] and lipoic acid [65] oligo(3-hydroxybutyrate) (OHB) conjugates. The ESI-CID spectra were acquired on Na^+^ adducts of the pesticide- and lipoic acid-OHB conjugates. Inspection of the experimental data clearly proved that the initiators, (4-chloro-2-methylphenoxy)acetate or (2,4-dichlorophenoxy)acetate, and the lipoic acid, respectively, were linked by covalent bonds to the OHB chains. Maksymiak et al. reported the synthesis of a biodegradable delivery system of p-coumaric acid (p-CA). OHB conjugates with p-CA (p-CA-OHB) were prepared by anionic ROO of β-butyrolactone and using p-CA potassium salt as initiator. The structure of the bioconjugate and the presence of side reaction products were checked by ESI-MS/MS. The ESI-CID mass spectrum of the Na^+^ adduct of p-CA-OHB with nine 3-HB units (*m*/*z* 961) showed two series of product ions originating from a β-H rearrangement. The product ions detected at *m*/*z* 797, 711, 625, 539, 453, 367, and 281, due to 3-HB oligomers bearing carboxyl and 4-hydroxy cinnamate end groups, originated from p-CA linked to the OHB chain. The complementary product ions at *m*/*z* 875, 789, 703, 617, 531, 445, and 359 were assigned to oligo-3-HB chains with crotonate and -COOH end groups. Therefore, the fragment ion at *m*/*z* 797 was due to the oligomer ended by crotonate and -COOH end groups due to the loss of p-coumaric acid (164 Da), whereas the ion at *m*/*z* 875 corresponded to the oligomer terminated by -COOH and 4-hydroxy cinnamate end groups, produced by the removal of crotonic acid. The MS/MS data confirmed the success of the synthetic procedure, showing that the selected oligomeric ions were constituted by 3-HB units and 4-hydroxy cinnamate and -COOH end groups [67]. Interestingly, Peptu et al. used cyclodextrins (CDs) for the ROO of L-lactide (LA) to prepare biodegradable systems with potential applications in the pharmaceutical field. The obtained CD isomeric mixtures were characterized by traditional techniques (GPC and NMR) and soft MS methods (ESI Q-TOF and MALDI TOF MS/MS) to determine the average number of monomeric units connected to the CDs and the structure of the derivatives. Fragmentation studies by MS/MS allowed proving the structure of the CD-LA, confirming the success of the synthetic procedure [80].

**Figure 8:**
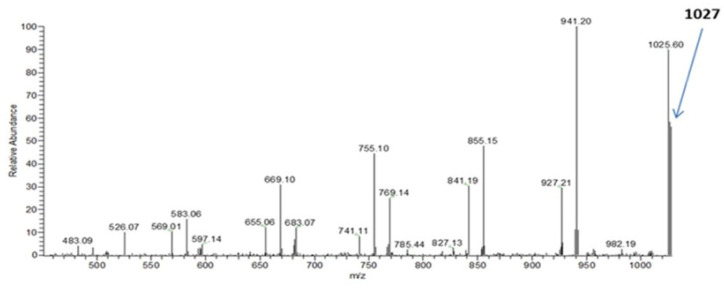
The ESI-MS/MS spectrum (positive-ion mode) of the PHA oligomers’ parent ion at *m*/*z* 1027 (reprinted from [125]).

Numerous studies have been focused on the ESI-MS/MS analysis of polyhydroxyalkanoates (PHA), a family of biodegradable and biocompatible polymers produced by some strains of bacteria in certain conditions [55,70,71,72,74,75,81,83,121]. Kowalczuk and Adamus carried out numerous investigations and reviewed the literature on MS and MS/MS procedures for the elucidation of the structure of microbial PHA, their synthetic analogs and degradation products [15]. They used the transesterification reaction of PHA as an easy route for the preparation of delivery systems based on bioactive compounds with -COOH or -OH groups. The structures of functionalized PHAs were established by ESI -MS/MS and ^1^H NMR [70,71,72,75]. PHA conjugates were obtained by a transesterification reaction with tyrosol, a phenolic bioactive compound with a -OH group present in a variety of natural sources. Poly(3-hydroxybutyrate-*co*-4-hydroxybutyrate) (P(3HB-*co*-4HB)) and poly-γ-glutamic acid (γ-PGA) were chosen as biodegradable polyester and polyamide, respectively. The syntheses were carried out in melt and catalyzed by 4-toluenesulfonic acid monohydrate. ESI-MS/MS confirmed the structure of conjugates in which bioactive compounds were covalently bonded to the PHA chains. The Authors displayed that the transesterification of P(3HB-*co*-4HB) with tyrosol produced oligomers with one bioactive molecule attached to the oligomer chain. On the contrary, the esterification of γ-PGA with tyrosol-originated conjugates with enhanced quantities of biologically active moieties along the backbone [71]. Similarly, Maksymiak et al. checked by ESI-CID and ^1^H NMR the functionalization with antioxidants used in cosmetics covalently linked as pendent groups along an oligomer backbone. Homo- and co-oligoesters were obtained via anionic ROO of p-methoxybenzoyloxymethylpropiolactone initiated by p-anisic acid sodium salt. Again, an analytical protocol based on MS/MS was developed for a detailed structural characterization of these bioactive (co)oligoesters [72]. With a view to a circular economy, Johnston et al. assessed by NMR and ESI-MS/MS the molecular structures of microbial PHA synthesized using, as a carbon source, waste polyethylene (PE) via non-oxygenated PE wax [74] and waste polystyrene (PS) pieces obtained using oxidative degradation [81]. In both studies, ESI-MS and fragmentation analysis by MS/MS was highly informative on end groups and co-monomeric unit structures and compositions. Very recently, Ekere et al. reported a new approach for recycling LDPE from Tetra Pak^®^ waste to synthesize PHAs using *Cupriavidus necator* as a foremost cheap option to the other recycling methods. The same research group previously depicted the exploitation of oxidized polypropylene (PP) [122] and oxidized PE wax (O-PEW) [123] as alternative carbon sources for PHA production, as well as a recycling approach for controlled oxidative fragmentation of LDPE plastics by *Cupriavidus necator* [124]. In all these interesting studies, the detailed structural assessment of the microbial PHA obtained was performed by ESI-MS/MS. The *Cupriavidus necator* is genetically stable and consistently stands out for its capacity to synthesize PHA from numerous carbon sources, such as sugars and fatty acids [124]. Thus, it was selected as a biocatalyst to prepare value-added PHA by LDPE portions in TetraPak^®^ waste. Again, ESI-MS and tandem MS were exploited for an exhaustive structural characterization of the PHA sample obtained from partial degradation of the PHA polymer extracted. The (+) ESI-MS spectrum of the selected PHA oligomer showed singly charged ions with a mass difference of 86 Da (i.e., the molecular mass of 3-hydroxybutyrate (3-HB) units) and different degrees of oligomerization and composition. The most abundant ions were due to the K^+^ adducts of PHB oligomers bearing crotonate and carboxylic end groups. Additional series of ions shifted by 14 to the most abundant ones were also detected. For further proof of the correct interpretation of the individual structures, ESI-MS/MS was performed on the precursor ions at *m*/*z* 1027 (Figure 8) [125].

Three series of product ions originated from the dissociation of the ion at *m*/*z* 1027. The first one (*m*/*z* 941, 855, 769, 683) corresponded to the K^+^ adducts of PHB oligomers [122]. The second series detected at *m*/*z* 927 and 841 originated from the removal of 2-pentenoic acid (100 Da) and showed the presence of 3-hydroxyvalerate and 3-HB units. The third smallest series of product ions (*m*/*z* 827, 741, 655, 569, and 483) was due to a loss of 2-hexenoic acid (114 Da), suggesting the presence of HH units along the PHA backbone. Consequently, according to the interpretation of MS/MS spectra, it was presumed that the ions at *m*/*z* 1027 were K^+^ adducts of the PHA oligomer bearing -COOH and unsaturated end groups, with three randomly distributed HV units or one HV unit and one HH unit with the structure ([HB8HV3 + K]^+^) or ([HB9HV HH + K]^+^), respectively. Nevertheless, NMR analysis did not confirm the presence of HH units [125].

In the literature, several papers have been published about the ESI-MS/MS analysis of biodegradable copolymers [51,53,55,57,58,61,69,71,72,74,77,81,121,126], compared to the limited number of publications on MALDI-MS/MS. Again, CID has been the mainly used fragmentation technique [59,62,77,83] (Table 1). Terrier et al. investigated NH_4_^+^ adducts of a series of linear triblock and glycerol-derivative block copolyesters by ESI CID under low-energy conditions. They preliminarily performed MS/MS analysis of the homopolymers and suggested a possible fragmentation mechanism by identifying the structure of the product ions. The Authors were able to differentiate between copolyesters with the same composition but with inversed block sequences. They showed that insights about block lengths could be easily found [57]. Adamus examined two random biodegradable copolyesters by ESI ion trap MS and tandem MS. The MS/MS analysis provided a comprehensive characterization of the copolymer samples, including the structure of the end groups (-OH and -COOH) and the co-monomeric unit distribution within the copolyester chains [58]. Duale et al. investigated the boric acid catalyzed homopolymerization and copolymerization of δ-valerolactone initiated by ethylene glycol and using 6-methyl-ε-caprolactone (mCL) as a comonomer P(dVL-co-mCL) [55,82]. The synthesized telechelic copolymers were analyzed by ^1^H NMR and ESI-MS to confirm, respectively, their chemical structures and composition. Again, ESI tandem MS was preliminarily utilized to establish the fragmentation pattern. Then, structural studies settled the formation of random linear copolymer chains consisting of different repeating units. The -OH groups at both copolymer ends were discovered by MS/MS experiments. The results of the fragmentation studies of P(dVL-co-mCL) with two mCL units undoubtedly revealed that the mCL units were included along with the copolymer chains [55].

### 3.3. Degradation Investigations

The development of biodegradable, biocompatible, and bioresorbable polymers and items must include the whole lifetime cycle, during which degradation can take place. It has to consider issues about synthesis, processing, manufacturing, sterilization processes, shelf life, and end-life options. Polymer degradation is a relevant feature with applicative drawbacks or, in some cases, advantages. It can be due to irradiation, heat, microorganisms, or chemicals and induced by more than one environmental factor [14,19,28]. Whatever the cause of the degradation, usually, products with distinctive functional and/or chain ends are generated and monitored by various analytical techniques (MS; NMR; Fourier transform infrared spectroscopy, FTIR) to obtain information on the deterioration mechanism. MS can provide both qualitative and quantitative insights into degradation compounds and macromolecular structural changes induced by various deterioration causes. The identification of degradation product structure can be helpful in showing indiscriminate, preferential, or selective enzymatic or hydrolytic bond cleavage [113,114,115,116,117,119,128]. Degradation phenomena yield permanent structural changes that are commonly undesirable as far as valuable properties are impaired. In some cases, degradation processes are useful and required, after their lifetime, as in biodegradable polymers. In addition, they are induced for structure identification, such as in pyrolysis-GC/MS (Py-GC/MS) [22,35,134] or as pre-treatment to reduce the MW for successive analyses.

**Figure 9:**
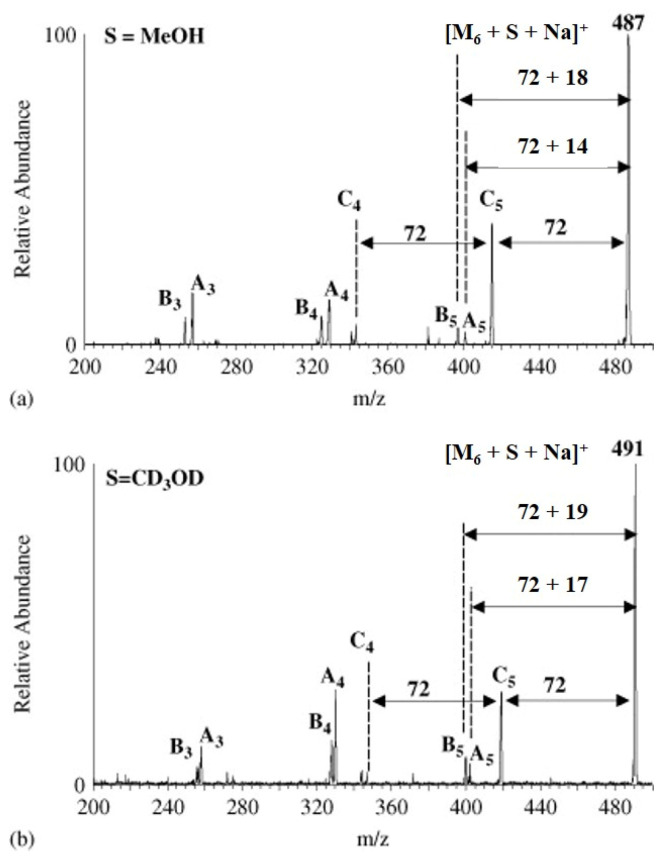
MS/MS spectra of [M_6_ + S + Na]^+^ *m*/*z* 487 (S = MeOH) (**a**) and *m*/*z* 491 (S = CD_3_OD) (**b**) following solvolysis of uniform CPLA (n = 6) (reprinted from [114] with kind permission of Wiley).

**Scheme 4:**
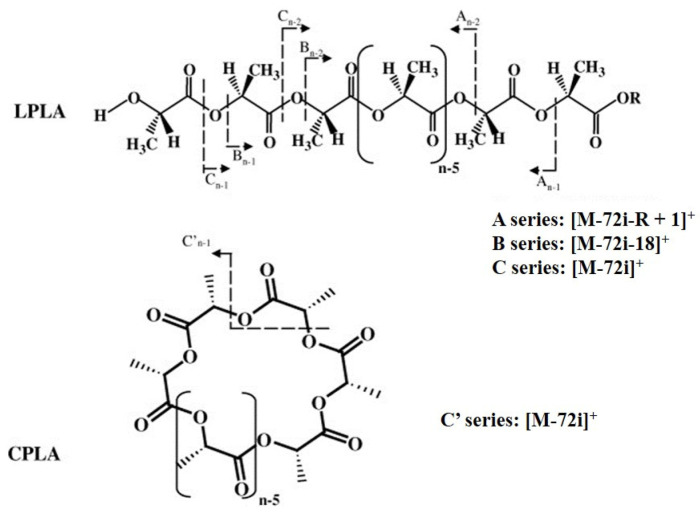
Chemical structures of linear (LPLA) and cyclic polylactic acid (CPLA), and definitions of ion series A, B, C, and C′ in the MS/MS spectra (reprinted from [114] with kind permission of Wiley).

The advancement and the nature of hydrolytic or enzymatic degradation products have been explored by various techniques, among which are soft MS methods and tandem MS [111,112,113,114,115,116,117,118,119,120]. ESI-MS and MS/MS are adopted more extensively and helpfully for characterizing low-MM oligomers bearing different end-group structures. The possibility of being interfaced with chromatographic techniques, i.e., high-performance liquid chromatography (HPLC), is a significant advantage that ESI-MS holds. Fragmentation studies are preliminarily carried out to proceed with additional investigations [114,115]. Osaka et al. investigated in detail the fragmentation behaviors and degradation by methanolysis of linear (LPLA) and cyclic (CPLA) PLA oligomers by ESI-MS and MS/MS. ESI tandem MS generated in LPLA and CPLA three types and one kind of series of fragment ions, respectively. Solvolysis was fully achievable but did not promptly occur in the presence of water, even in small amounts. In the MS/MS spectrum of the solvolysis ions [M_6_ + MeOH + Na]^+^ at *m*/*z* 487, three types of fragment ions (A, B, and C series) were detected (Figure 9a). The Authors deduced that if the *m*/*z* 487 ion was due to a noncovalent complex between CPLA (n = 6) and methanol (MeOH), the loss of MeOH (32 Da) would be easily revealed. The non-appearance of signals at *m*/*z* 487 -32 suggested that the [M_6_ + MeOH + Na]^+^ ion corresponded to a linear PLA due to solvolysis. Similarly, three ion series (labeled A, B, and C) were revealed in the tandem MS spectrum of [M_6_ + CD_3_OD + Na]^+^ (*m*/*z* 491) of the CD_3_OD degradation products (Figure 9b). The spectrum was equivalent to that acquired when the methanol was replaced by CD_3_OD. The results suggested that methanolysis produced smaller LPLA by both LPLA and CPLA. In fact, C series ions were detected in both the MS/MS spectra of [M_6_ + S + Na]^+^ (S = MeOH and CD_3_OD) (Figure 9), showing that it derived from the removal of a lactic acid molecule not belonging to the LPLA end group. The dissociation of C ions was supposed to imply a rearrangement of the H atom at the ω end group (Scheme 4) [114].

The hydrolytic degradation of biodegradable homo and copolyesters of 1,5-dioxepan-2-one (DXO), CL, and cross-linked polyester-ether networks was monitored by ESI-MS and tandem MS. A selective scission was observed for DXO oligomers, with a higher propensity for ether with respect to ester bond breakage under the trial conditions. ESI tandem MS disclosed the effect of hydrophilicity on the water-soluble degradation products of both homo and copolyesters of DXO and CL. Indeed, the degradation product profile of the DXO-CL-DXO triblock copolymer largely involved the more hydrophilic DXO-based oligomers, showing favored hydrolysis of DXO blocks, also proved by NMR [115,116]. The biodegradation behavior of composites based on P(3HB-*co*-4HB) and wood flour (10, 20, and 30 wt. %) as a filler (a cellulose-based waste) has been studied, under laboratory composting conditions, by Musioł et al. Abiotic degradation tests in water and buffer solution at 70 °C were carried out as well. The degradation products were analyzed by ESI tandem MS. In the ESI-MS spectra of P(3HB-*co*-4HB) oligomers released in water after 70 days of incubation, mainly two series of Na^+^-charged ions with different degrees of oligomerization were detected. Nevertheless, the two monomer units, 3-HB and 4-HB, had the same exact MM (86 Da) and cannot be distinguished by MS. As a result, ESI tandem MS was utilized to verify the chemical structures of the degradation products, differentiating isobaric structures. For all the analyzed samples, the same degradation products were established [120].

HPLC/ESI-MS has been displayed to be a valuable method for the analysis of enzymatic degradation products of biodegradable polymers [111,112,113,117,118,119]. Woo et al. showed that ciprofloxacin (a fluoroquinolone antibiotic) could be polymerized into the backbone of PCL and that the polymer can be degraded by cholesterol esterase (an inflammatory cell-derived enzyme). They employed HPLC/ESI-MS and MS/MS offline analysis to check the release of ciprofloxacin in response to inflammatory-related enzymes. Multiple degradation products were separated by HPLC and analyzed by MS/MS. They included ciprofloxacin and its derivatives, among which ciprofloxacin bonded to fragments of PCL and HDI that did not display antimicrobial activity [111]. Water-soluble monomers and co-oligomers from the lipase-assisted hydrolysis of synthetic poly(butylene succinate-*co*-butylene sebacate) (P(BSu-*co*-BSe)) and P(BSu-*co*-BA) samples were analyzed by HPLC/ESI-MS. The optimization of chromatographic parameters allowed the separation of isobaric co-oligomers, which were different just for the comonomer sequence. These degradation products, with identical monomer composition and MM but diverse sequences, were differentiated by HPLC/ESI-MS/MS online analysis. Remarkably, the MS/MS data showed a favored hydrolytic bond scission produced by the enzymes. The selected lipases preferred breaking sebacic ester bonds in P(BSu-*co*-BSe) copolymers, while succinic ester bonds seemed to be hydrolyzed quicker than adipic ester ones in P(BSu-*co*-BA) copolyesters. These conclusions were further confirmed by ^1^H NMR analysis [113]. With a similar methodology, Pulkkinen et al. accomplished the HPLC/ESI-MS/MS online analysis of the enzymatic degradation products of 2,2(-bis(2-oxazoline)-linked PCL) (PCL-O). Polymer films were prepared by a solvent cast, and their enzymatic degradation was evaluated in simulated intestinal fluid (phosphate buffer, pH 7.5, 1% pancreatin). The enzymatic degradation of the polymer produced a wide variety of water-soluble oligomers. Again, optimization of the HPLC parameters resulted in effective separation of the oligomers that were clearly identified by tandem MS. According to the structures of the PCL-O degradation products, ester bonds were confirmed the most sensitive to enzymatic degradation. In fact, specific structures of the PCL-O oligomers predictably showed that pancreatic enzymes cleaved mostly ester bonds and were usually not able to break down the amide ones. Accordingly, pancreatic lipase was mainly responsible for the enzymatic erosion of the PCL-O films [117]. Moreover, LC/ESI-MS/MS provided detailed structural information on a commercial poly(butyleneadipate-*co*-butyleneterephthalate) (PBAT) and its partial degradation products attained under alkaline conditions. LC/MS and LC-MS/MS showed the presence of cyclic structures in the virgin samples, which fully disappeared after degradation. Additionally, the occurrence of methanol transesterification reaction in the degradation process was highlighted. MS/MS on the first ^13^C isotope peak was helpful in establishing the elemental composition of the fragment ions and the end group structures. The method was proposed as an alternative for high mass accuracy tandem MS experiments. Finally, sequence distribution was disclosed for copolymeric structures [118].

### 3.4. Bioresorbable Polymers and DDS Studies by LC-ESI Tandem MS

Bioresorbable polymers are biodegradable systems that can be absorbed by the body through hydrolytic or enzymatic reactions. Their chemical–physical properties, biocompatibility, and adjustable degradation rates make them fitting for several applications such as DDS. Bioresorbable and biocompatible synthetic polymers are mainly polyesters and include PLA and its derivatives, poly(glycolide) (PGA), their copolymers (PLGA), PCL, PEG, polydioxanone, etc. PLA and its copolymers are the most extensively utilized [135]. They are widely employed in formulating nanoparticles, appealing drug delivery vehicles, and combining biocompatibility with enhanced drug bioavailability, half-life, and toxicity profile of nanocarriers. The development of these systems offered new approaches to fighting against diseases [136]. PLGA copolymers are approved by FDA and are the most broadly studied for controlled delivery systems. Modifying the monomer composition and MW, drug release can be tuned over several days to months [137].

Chromatographic separation combined with tandem MS has been more and more recurrently used in the assessment of biodegradable DDS, such as microspheres, micelles, or nanoparticles, measuring drug loading or concentration [97,100,101,102,103,104,105,106,107,108,109,111,112] and monitoring drugs or hormone release, in vitro and in vivo [89,90,91,95,96,102,110,111]. Recently, Ultra-HPLC (UHPLC) with MS/MS methods has been developed, attracting considerable interest in pharmacokinetic studies for its high sensitivity, accuracy, and precision. They provided an exhaustive characterization of DDS fate and pharmacokinetic behavior after administration [98,103,104,105,106,107,110] and were able to check the fate of pharmaceutical polymeric excipients [106,108]. Remarkably, a quantitative determination of CPLA oligomers in serum has been accomplished by direct injection LC-MS/MS. PLA is used in both pharmaceutical and surgical devices, and CPLA by-product may be introduced into the human body as an unwanted impurity. In particular, the Authors found by LC-MS that the CPLA heptamer (CPLA-7) bonded hard with serum proteins and that only 62% of CPLA-7 was regained after normal deproteination. They pretreated the serum through a passage in the bovine serum albumin (BSA)-coated chromatographic column. Then, the serum was directly introduced into an LC-MS/MS apparatus, and the recovery of CPLA-7 was improved to 84% [60].

**Figure 10:**
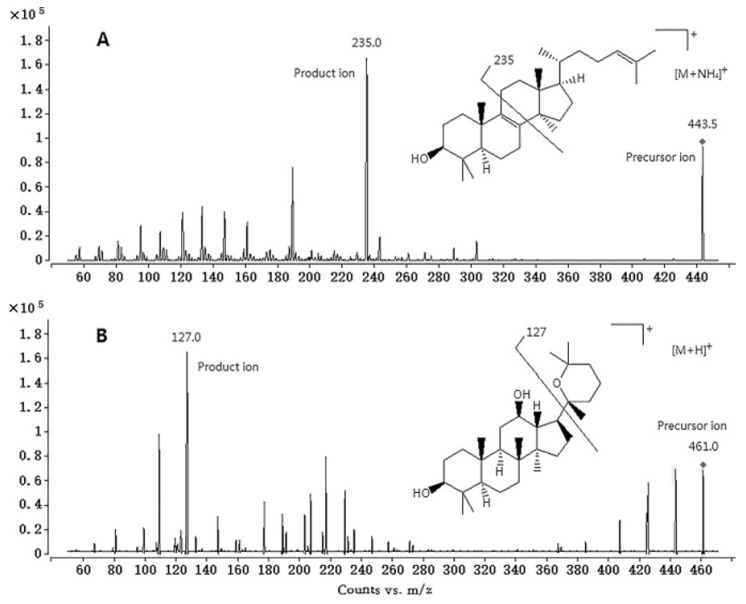
Product ions and structures of (**A**) lanosterol and (**B**) IS (reprinted from [110] with kind permission of Elsevier).

LC-MS/MS is more commonly used in the study of small molecular drugs. The main question for the quantitative evaluation of polymers by LC-MS/MS is principally related to their polydispersity. However, in recent times, great progress has been performed in the bioanalysis of polymers by LC-MS/MS. Numerous MS strategies, such as selected ion monitoring (SIM), multiple reaction monitoring (MRM), selected reaction monitoring (SRM), in-source CID, and MS^ALL^, have been strategically employed to overcome this challenge in the pharmaceutical polymer analysis (i.e., PEG and PLA). LC-MS/MS is among the innovative bioanalysis techniques for tracking the in vivo fate of nano DDS (NDDSs). NDDSs are designed ad hoc as carriers for the delivery of functional pharmaceutical ingredients to their target sites with various advantages. Subsequently, the administration of the polymeric components of NDDSs may undergo disassembly, distribution, metabolism, and excretion. Nowadays, LC-MS/MS is currently used for in vivo polymer quantitation NDDSs [108]. Perteghella et al. included an anticancer drug, paclitaxel (PTX), in silk fibroin nanoparticles (SFNs) and developed a plain and reliable method based on reversed-phase LC-MS/MS (rp-UHPLC-MS/MS) to quantify the PTX loaded in SFNs. ESI tandem MS and the SRM mode were employed. The transitions of the mass-to-charge ratio at 854.1 → 509.3 and *m*/*z* 854.1 → 286.2 were selected, respectively, for the qualitative and quantitative estimation of PTX applying the calibration curve from the PTX standard solutions to quantify the loaded PTX [100]. Lanosterol is a potential drug for cataracts, and it was tested in a thermogel formulation based on a poly-(DL-lactic acid-co-glycolic acid)–poly(ethylene glycol)–poly-(DL-lactic acid-co-glycolic acid) (PLGA–PEG–PLGA) by Ly et al. It was quantified by UHPLC–ESI-MS/MS in the vitreous humor of rabbits after ocular administration of the formulation. The analyte was quantified in positive ionization by MS using MRM mode. The [M + NH_4_]^+^ and [M + H]^+^ were the major ions for lanosterol and internal standard (IS), respectively, in the Q1 spectrum and were selected as the precursor ions. The mass transitions *m*/*z* 443.5 → 235 and *m*/*z* 461 → 127 were chosen to determine the analyte and IS, respectively. Product ions and structures of lanosterol, as well as IS, are reported in Figure 10. The MS/MS working parameters were optimized to enhance the response for the lanosterol, avoiding endogenous interference at the retention time of the drug (4.1 min) and IS (3.7 min). The Authors reported a run time of 5 min per sample, which means more than 200 samples per day. This simple, rapid, sensitive, and specifically validated method was also exploited to investigate vitreous samples of white rabbits from New Zealand for pharmacokinetic studies. The experimental data furnished valuable information on the pharmacological action mechanism of lanosterol and for cataract treatment [110].

UHPLC tandem MS combined with in-source CID was used by Shi et al. to develop a specific and sensitive analytical approach to quantify amphiphilic block PEG-PLA copolymers in plasma. A representative compound (mPEG2000-PDLLA2500-COOH) was dissociated from the source. The series of product ions, particularly PLA-specific and PEG-diagnostic fragment ions, were further dissociated into distinctive ions in the collision cell. After all, the ion transition at *m*/*z* 505.0 → 217.0 was selected for the quantitative determination of mPEG2000-PDLLA2500-COOH. The fragment ions related to PEG were MRM transition examined for PEG-PLA. Then, the method was successfully employed in the pharmacokinetic study of the model compound in rats. Interestingly, it could be potentially expanded to the study of other pharmaceutical polymer excipients [106]. PEG is one of the most important synthetic polymers used in the pharmaceutical field. It is extensively employed as a stabilizer and solubilizer in block copolymers, conjugated with drugs (PEGylation) and DDS (i.e., micelles and nanoparticles). Ashiru et al. established a specific LC-MS/MS procedure for the quantitative determination in biological samples of PEG 400, used as an excipient in oral formulations. A direct injection ESI SIM MS method and external calibration were employed. This SIM method had a restricted selectivity, being susceptible to interference from endogenous substances, but the limit of quantification was greater than that of the flow injection MS method [92]. Bhaskar et al. analyzed by the MRM a PEG400 in rat plasma with improved selectivity and signal-to-noise ratio. Telmisartan was selected as the internal standard, and PEG400 was separated from rat plasma with acetonitrile. The nine most abundant ions identified for PEG400 and the associated product ion at *m*/*z* 89 were selected for MRM in ESI mode. The peak areas of the analyte were then summed up to estimate the total concentration of PEG400 in plasma with a very low quantification limit. However, this strategy may be suitable for low molar mass (MM) PEG samples. In fact, mass spectra of high MM PEGs include a broad extent of homologs and multicharged ions, among which only a fraction can be verified by MRM that, in such case, would be unsatisfactory for quantitation [93]. Therefore, Warrack et al. elaborated an analytical strategy for the quantitative analysis of high MM PEG (1.4–40 kDa) in biological samples without complex preliminary sample preparation. The reported method aimed to establish whether high-MM PEG was cut in vivo to lower-MM PEG species. In-source CID was combined with MRM or SIM MS and then used to monitor specific PEG fragment ions. Estimation of MM was achieved by the retention times in reversed-phase LC. Nonetheless, the detection was restricted by inadequate dissociation in the ion source, which finally reduced the sensitivity of the successive MRM scan [94]. Thus, Zhou et al. designed an MS^ALL^-based strategy with a higher fragmentation yield for the quantitative determination of PEG by LC-Q-TOF MS, a hybrid MS equipped with two quadrupoles in sequence (Q1, Q2), and a high-resolution TOF mass analyzer. In the MS^ALL^ scan mode, all precursor ions are selected by Q1 and then dissociated in Q2, with all fragment ions separated by the TOF analyzer. A specific PEG product ion was noticed to be shared with all linear PEGs and permitted the quantification in rat plasma with high sensitivity, outstanding linearity, and reproducibility. The developed test was effectively extended to pharmacokinetic studies, showing valuable potentiality in other applications for different pharmaceutical polymers [99,109].

## 4. Conclusions

The use of biodegradable and biobased plastic materials can be really beneficial in the desired and foreseen conversion towards a circular economy. In this view, these polymers are an increasingly valuable class of materials in various fields. However, the cost and performance are not as functional as those of traditional plastic materials. In fact, the design and development of biodegradable polymers with appropriate properties for different usages and, simultaneously, with harmful degradation products is still a challenge.

For all types of materials, characterization is essential for understanding the structure–property relationships and testing the suitability for a particular purpose. Analytical methods and strategies based on single-stage MS have been successfully utilized for characterization as well as degradation features of polymeric materials, including biodegradable ones. Nevertheless, single-stage mass analysis is not always sufficient to provide unequivocally the required structural information. In such instances, MS/MS can be a useful support. It can help to discern isobaric and isomeric species, supply additional data on chain end or in-chain substituents, discriminate cyclic and linear polymers, and establish macromolecular bond connections, sequences, and architectures. Although tandem MS can be greatly informative, it has been less frequently used than single-stage MS, reasonably for time-consuming data processing and interpretation. In any case, preliminary fragmentation studies are required for the comprehension of tandem mass spectra. In fact, knowledge of the dissociation pathways of the selected precursor ions allows for assuming reliable assignments from the fragment ions detected in tandem MS spectra.

The hereby-reviewed literature, focused on MALDI and ESI-MS/MS, highlighted that both techniques had been effectively and successfully used in biodegradable polymer analyses. Fragmentation and mechanistic investigations, structural and architectural studies, biopharmaceutical and drug delivery systems analyses, and polymer degradation monitoring have been carried out mainly on biodegradable polyesters. Through inspection of the MS and MS/MS mass spectra, acquired essentially in positive ion mode, noteworthy insights on biodegradable macromolecular architectures and structural details, end-group functionalities, copolymer composition, bond and sequence distribution, pharmacokinetics, the fate of polymeric excipients, as well as DDS and the profile of degradation products have been achieved. In most cases, traditional analytical methods (i.e., NMR or FTIR, Table 1) or LC separation were used as well to validate or support the results. Moreover, IM-MS/MS has fruitfully provided detailed information on structural differences, particularly macromolecular isomers and isobars, in biodegradable polymer samples.

Overall, ESI-MS/MS has been more frequently employed than MALDI tandem MS in biodegradable polymer analyses. CID is the most adopted activation method, even though it tends to induce multiple or sequential bond cleavages, providing more complex spectra than in the less-used ETD experiments. In most of the papers, both techniques are used in the structural characterization, i.e., to check the success of the polymerization reaction and in DDS studies. Limited research has addressed the degradation studies of biodegradable polymers. ESI-MS/MS has proved to be a valuable and elective method for both qualitative and quantitative information on water-soluble monomers and oligomers originating from degradation and monitoring drug release or pharmacokinetic studies. Its success and wider applications undoubtedly are settled in the advantage of being easily interfaced with solution-based separation techniques, such as HPLC, for separating complex mixtures.

## Figures and Tables

**Table 1:** Summary of MALDI and ESI tandem MS in microbial and synthetic biodegradable polymers.

Polymers	MS/MS Techniques	Other Methods	Information Acquired by MS/MS	Ref.
mPEG	MALDI–CID	SEC; ^1^H NMR	Nature of end groups and architectural modifications	[44]
PEAm	MALDI–CID	PSD-MALDI-TOF	Fragmentation mechanisms and ester/amide sequences	[47]
PBA	MALDI–CID	/	Fragmentation mechanisms	[48]
PEAm	MALDI–CID	/	Fragmentation mechanisms and ester/amide sequences	[49]
PLA	ESI–CID	/	Mechanistic study	[50]
Poly(ethylene adipate) and PBA, PLA and copolymers	ESI–CAD/ETD	/	CAD/ETD comparison	[51]
PBSu	MALDI–CID	/	Fragmentation mechanisms	[52]
PCL, PTHF and copolymer	ESI–CAD/ETD	/	CAD/ETD comparison	[53]
PEAm	MALDI–CID	/	Fragmentation mechanisms and ester/amide sequences	[54]
PdVL and P(dVL-co-mCL)	ESI–CID	^1^H NMR	Fragmentation mechanisms, end groups and molecular structures	[55]
Hyperbranched PEAm	ESI-FT-ICR–CAD	/	Fragmentation mechanisms, isomeric structures and composition	[56]
PEO, PPO, linear triblock and glycerol derivative diblock copolyethers	ESI–CID	/	Fragmentation mechanisms, block lengths and sequences	[57]
P[(R,S)-3HB-*co*-LA]	ESI–CID	GPC; ^1^H NMR	Fragmentation mechanisms, molecular structures, end groups and sequences	[58]
PEO-*co*-PPO	MALDI–CID	LAC–MALDI offline; software tool	Differentiation between isobaric structures	[59]
CPLA oligomers	LC-ESI–CID	/	Quantitative determination in serum	[60]
Poly[(*R*,*S*)3-hydroxy-4-ethoxybutyrate-*co*-(*R*,*S*)-3-hydroxybutyrate)], Poly[(*R*,*S*)-3-hydroxybutyrate)-*block*-(*R*,*S*)-3-hydroxy-4-ethoxybutyrate)]	ESI–CID	SEC; FT-IR; ^1^H NMR	Molecular structures and sequence distribution	[61]
Hexanediol-neopentylglycol-adipic acid copolyesters	MALDI–CID	/	Fragmentation mechanism, differentiation between cyclic and linear oligomers	[62]
Phenoxycarboxylic acid–oligo(3-hydroxybutyrate) conjugates	ESI–CID	GPC; FT-IR; ^1^H NMR	Confirmation of structure assignment and functionalization	[63]
PLA with different isomeric end groups	ESI–CID	/	Isomeric end-group distinction	[64]
Lipoic acid–oligo-(3-HB) conjugates	ESI–CID	GPC; HPLC-ESI/MS; ^1^H NMR; FTIR	Structural characterization	[65]
p-coumaric acid–oligo(3-HB) conjugates	ESI–CID	GPC; FT-IR; ^1^H NMR	Confirmation of structure assignment and functionalization	[67]
CPLA	ESI–CID	SEC; MALDI-MS; ^1^H NMR	Detection of architectural impurity	[68]
Pesticide PHAs conjugates	ESI–CID	GPC; ^1^H NMR	Confirmation of structure assignment and functionalization	[70]
Tyrosol-P(3HB-*co*-4HB) and γ-PGA conjugates	ESI–CID	GPC; ^1^H NMR	Structural characterization and successful polymerization	[71]
(*p*-AA-CH_2_-HP)_n_, p-AA-CH_2_-HP)_m_/HB_n_	ESI–CID	SEC; FT-IR; ^1^H NMR	Fragmentation mechanism, molecular structures, end groups and copolymer composition	[72]
Microbial PHAs	ESI–CID	GPC; FT-IR; ^1^H NMR	Structure identification	[74]
Peptides-PEG conjugates	MALDI–CID; ESI–CAD/ETD, ESI-IM-MS CAD	CD	Elucidation of alanine-rich polypeptides sequence and conformation	[76,78]
PPM, PPF	MALDI–CID; ESI–CAD/ETD, ESI-IM-MS CAD	SEC, Molecular modeling	Composition, end groups and chain sequence differentiate the isomeric PPM and PPF	[77]
CDLA	MALDI–LID, ESI–CID	GPC; ^1^H NMR	Structural architecture, influence of the cationization agent on fragmentation profiles, average length of the PLA chains attached to the CD	[80]
Microbial PHAs	ESI–CID	GPC; FT-IR; ^1^H NMR, TGA	Structure identification	[81]
PdVL	ESI–CID	GPC; MALDI-MS; ^1^H NMR	Molecular structure, end groups	[82]
P3HB-*b*-3HV	MALDI–CID	GPC; ESI-MS; ^1^H and ^13^C NMR, solution-state NMR; DSC	Fragmentation mechanisms, presence of some randomness	[83]
PLGA	ESI–CID	SEC; NMR; SEC/MALDI-MS offline; reactive-SALDI; Kendrick analysis	Structure and end groups	[84]
Peptides-polymers conjugates	MALDI–CID; ESI–CAD/ETD, ESI-IM-MS CAD	Molecular modeling	Architectural microstructure	[85]
PC	ESI–ETD	^1^H and ^13^C NMR; MALDI-MS; DSC; TGA	Distribution of head-to-head, head-to-tail, and tail-to-tail regiochemistries	[86]
CDLA	MALDI–LID	GPC; ^1^H NMR	Structural architecture, interference of the solvents	[87]
CDCL	MALDI–LID	1D and 2D NMR	Structure confirmation	[88]

mPEG = substituted methoxy poly(ethylene glycol); PEAm = polyesteramides; PBA = poly(butylene adipate); PLA = polylactide; PBSu = poly(butylene succinate); PCL = polycaprolactone; PTHF = polytetrahydrofurane; PdVL = poly(δ-valerolactone); P(dVL-*co*-mCL) = random poly(δ-valerolactone-*co*-6-methyl-ε-caprolactone); PEO = poly(ethylene oxide); PPO = poly(propylene oxide); P[(R,S)-3HB-*co*-LA] = poly[(R,S)-3-hydroxybutyrate-*co*-L-lactide]; PEO-*co*-PPO = polyethylene oxide-polypropylene oxide copolymers; CPLA = cyclic PLA oligomers; 3HB = 3-hydroxybutyrate; PHAs = polyhydroxyalkanoates; P(3HB-*co*-4HB) = poly(3-hydroxybutyrate-*co*-4-hydroxybutyrate); γ-PGA = poly-γ-glutamic acid; (p-AA-CH_2_-HP)_n_ = oligo(3-hydroxy-3-(4-methoxybenzoyloxymethyl)propionate); (p-AA-CH_2_-HP)_m_/HB_n_ = oligo[(3-hydroxy-3-(4-methoxybenzoyloxymethyl)propionate)-*co*-(3-hydroxybutyrate)]; PEG = poly(ethylene glycol); PPM = poly(propylene maleate); PPF = poly(propylene fumarate); CDLA = cyclodextrin (CD) oligolactide derivatives; P3HB-*b*-3HV = poly-3-hydroxybutyrate-*b*-3-hydroxyvalerate block copolymers; PLGA = poly(lactide-*co*-glycolide); PC = polycarbonates; CDCL = cyclodextrin (CD) oligocaprolacton derivatives; MALDI = matrix-assisted laser desorption ionization; ESI = electrospray ionization; IM = ion mobility; CID = collision induced dissociation; CAD = collision activated dissociation; ETD = electron transfer dissociation; LC-ESI = liquid chromatography-ESI; FT-ICR = Fourier transform ion cyclotron resonance; LID = laser induced dissociation; SEC = size-exclusion chromatography; NMR = nuclear magnetic resonance; PSD = post source decay; TOF = time of flight; GPC = gel permeation chromatography; LAC = liquid adsorption chromatography; FT-IR = Fourier transform infrared spectroscopy; HPLC = high performance liquid chromatography; CD = circular dichroism; DSC = differential scanning calorimetry; TGA = thermogravimetry analysis; reactive-SALDI = surface-assisted laser desorption/ionization in a reactive mode.

## Data Availability

Not applicable.
